# Persistence of Epigenomic Effects After Recovery From Repeated Treatment With Two Nephrocarcinogens

**DOI:** 10.3389/fgene.2018.00558

**Published:** 2018-12-03

**Authors:** Alice Limonciel, Simone G. van Breda, Xiaoqi Jiang, Gregory D. Tredwell, Anja Wilmes, Lydia Aschauer, Alexandros P. Siskos, Agapios Sachinidis, Hector C. Keun, Annette Kopp-Schneider, Theo M. de Kok, Jos C. S. Kleinjans, Paul Jennings

**Affiliations:** ^1^Division of Molecular and Computational Toxicology, Amsterdam Institute for Molecules, Medicines and Systems, Vrije Universiteit Amsterdam, Amsterdam, Netherlands; ^2^Division of Physiology, Department of Physiology and Medical Physics, Medical University of Innsbruck, Innsbruck, Austria; ^3^Department of Toxicogenomics, GROW-School for Oncology and Development Biology, Maastricht University Medical Center, Maastricht, Netherlands; ^4^Division of Biostatistics, German Cancer Research Center (DKFZ), Heidelberg, Germany; ^5^Division of Cancer, Department of Surgery and Cancer, Faculty of Medicine, Imperial College London, Hammersmith Hospital, London, United Kingdom; ^6^Department of Applied Mathematics, Research School of Physics and Engineering, Australian National University, Canberra, ACT, Australia; ^7^Brookes Innovation Hub, Orbit Discovery, Oxford, United Kingdom; ^8^Institute of Neurophysiology and Center for Molecular Medicine Cologne (CMMC), University of Cologne (UKK), Cologne, Germany

**Keywords:** recovery, persistence, epigenomics, stress responses, ochratoxin A, potassium bromate, nephrotoxicity, metabolomics

## Abstract

The discovery of the epigenetic regulation of transcription has provided a new source of mechanistic understanding to long lasting effects of chemicals. However, this information is still seldom exploited in a toxicological context and studies of chemical effect after washout remain rare. Here we studied the effects of two nephrocarcinogens on the human proximal tubule cell line RPTEC/TERT1 using high-content mRNA microarrays coupled with miRNA, histone acetylation (HA) and DNA methylation (DM) arrays and metabolomics during a 5-day repeat-dose exposure and 3 days after washout. The mycotoxin ochratoxin A (OTA) was chosen as a model compound for its known impact on HA and DM. The foremost effect observed was the modulation of thousands of mRNAs and histones by OTA during and after exposure. In comparison, the oxidant potassium bromate (KBrO_3_) had a milder impact on gene expression and epigenetics. However, there was no strong correlation between epigenetic modifications and mRNA changes with OTA while with KBrO_3_ the gene expression data correlated better with HA for both up- and down-regulated genes. Even when focusing on the genes with persistent epigenetic modifications after washout, only half were coupled to matching changes in gene expression induced by OTA, suggesting that while OTA causes a major effect on the two epigenetic mechanisms studied, these alone cannot explain its impact on gene expression. Mechanistic analysis confirmed the known activation of Nrf2 and p53 by KBrO_3_, while OTA inhibited most of the same genes, and genes involved in the unfolded protein response. A few miRNAs could be linked to these effects of OTA, albeit without clear contribution of epigenetics to the modulation of the pathways at large. Metabolomics revealed disturbances in amino acid balance, energy catabolism, nucleotide metabolism and polyamine metabolism with both chemicals. In conclusion, the large impact of OTA on transcription was confirmed at the mRNA level but also with two high-content epigenomic methodologies. Transcriptomic data confirmed the previously reported activation (by KBrO_3_) and inhibition (by OTA) of protective pathways. However, the integration of omic datasets suggested that HA and DM were not driving forces in the gene expression changes induced by either chemical.

## Introduction

Ochratoxin A (OTA) is a food contaminating mycotoxin, a nephrotoxin and a suspected renal carcinogen (Limonciel and Jennings, 2013). In Europe, the average daily intake of OTA has been estimated at 1 ng.kg^−1^ b.w. but exposures up to eight times higher have been reported (Schaaf et al., 2002; Clark and Snedeker, 2006). Its mechanism of toxicity remains elusive, with some studies suggesting genotoxicity, others suggesting epigenetic effects and yet others showing OTA-induced disturbances in the Nrf2 response to oxidative stress (Limonciel and Jennings, 2013; Vettorazzi et al., 2013). One striking effect of OTA is its very large impact on the transcriptome, affecting the expression of thousands of genes in both *in vitro* and *in vivo* settings (Jennings et al., 2012). Networks affected include genes involved in cytoskeleton organization, nucleosome regulation, transcription and translation, ubiquitination and cell cycle regulation. However, from the initiation of gene transcription to the splicing and maturation of mRNAs, a multitude of steps can alter gene expression and result in a disturbance of cellular homeostasis. Targeted mechanistic investigations have revealed that OTA perturbs the acetylation of proteins in general and of histones in particular. More specifically, OTA inhibited histone acetyltransferases (HATs) *in vitro* (Czakai et al., 2011) and enhanced the activity of histone deacetylases (HDACs) (Marin-Kuan et al., 2006), suggesting a global deacetylating effect. In rats, this toxin impacted the maturation of microRNAs (miRNAs) via a down-regulation of the expression of the genes encoding Dicer1 and Drosha (Dai et al., 2014). Thus, the large impact of OTA on gene expression could be due to a combination of factors including epigenetic modifications and differential miRNA regulation.

KBrO_3_ is an oxidiser historically manufactured for primary use in bread preparations and hair products (International Agency for Research on Cancer, 2018). While bromate is not known to form in nature, it has been shown to occur during drinking water ozonation. Numerous cases of acute human exposures have been reported, usually following voluntary ingestions or after accidental contamination of bread preparations with excessive amounts of KBrO_3_, causing nephrotoxicity and ototoxicity in children and adults (Campbell, 2006). The IARC classified KBrO3 as a possible carcinogen to humans as a consequence of the evidence found in rodents but in the absence of chronic exposure data in humans. In rodents, KBrO_3_ exposure resulted in reactive oxygen species production and a depletion of glutathione, involved in the protection against oxidative stress (Sai et al., 1992; Zhang et al., 2010) as well as DNA damage (Ballmaier and Epe, 2006) involving the formation of 8-OHdG (Kasai et al., 1987; Cho et al., 1993), micronuclei (Hayashi et al., 1988) and chromosomal aberrations (Ishidate et al., 1984; Fujie et al., 1988).

In the current study, we investigated the effects of OTA and KBrO_3_ on epigenetic modifications and miRNAs, and their potential link to the transcriptomic effects caused by the test chemicals. To this end, the global effects on mRNA and miRNA expression and epigenetic modifications (DNA methylation (DM) and histone acetylation (HA)) were integrated and compared in a human renal proximal tubule cell line (RPTEC/TERT1) exposed to the chemicals in a repeat-dose testing regime and after a recovery period of 3 days after treatment. In addition, we investigated the metabolomic profile of these cells during and after exposure to identify downstream dysfunctions in homeostasis regulation. The modulation of stress response pathways was also addressed within the mechanistic investigation, with a particular focus to the Nrf2 response to oxidative/alkylating stress and the activation of p53, another transcription factor widely known as a tumor suppressor for its role in the maintenance of DNA integrity in the presence of carcinogens, for which KBrO_3_ served as a positive control (Limonciel et al., 2012).

## Materials and Methods

### Chemicals

The two chemicals in study were purchased from Sigma-Aldrich (OTA, O1877 and KBrO_3_ P7332). All chemicals unless otherwise stated were purchased from Sigma and were of the highest grade available.

### Cell Culture

Under routine conditions, human proximal tubule RPTEC/TERT1 cells (Wieser et al., 2008) were cultured at 37°C in a 5% CO_2_ humidified atmosphere, fed 3 times a week and sub-cultured by trypsinisation. RPTEC/TERT1 cells were seeded onto 96-well cell culture plates (655180, Greiner) for concentration screening, PET 96-well E-plate VIEW cell culture plates (300600910, ACEA) for impedance measurements, 6-well cell culture plates (657160, Greiner) for mRNA and miRNA sample preparation, and on 10-cm cell culture dishes (831802, Sarstedt) for all other measurements. Cells were grown in hormonally defined medium (HDM) as previously described (Aschauer et al., 2013). Briefly, after confluence was reached, the cells were allowed to stabilize and form a contact-inhibited monolayer for ten days with feeding every 2–3 days. HDM consisted of a 1:1 mixture of Dulbecco’s modified Eagle’s medium (DMEM, Invitrogen, cat. no. 11966) and Ham’s F-12 nutrient mix (Invitrogen, cat. no. 21765) supplemented with 2 mM glutamax (Invitrogen, cat. no. 35050-038), 5 μg/mL insulin, 5 μg/mL transferrin and 5 ng/mL sodium selenite, 100 U/mL penicillin and 100 μg/mL streptomycin, 10 ng/mL epithelial growth factor and 36 ng/mL hydrocortisone.

Stocks of the test chemicals were prepared as follows. Five milligrams OTA were dissolved in DMEM/F-12 medium (without additives) to a 2.48 mM stock and further diluted to a 50× stock (6.5 μM) in DMEM/F-12. KBrO_3_ was directly dissolved to a 50X stock (40 mM) in the same aqueous solvent.

### Cell Viability and Cell Stress

**FIGURE 1:**
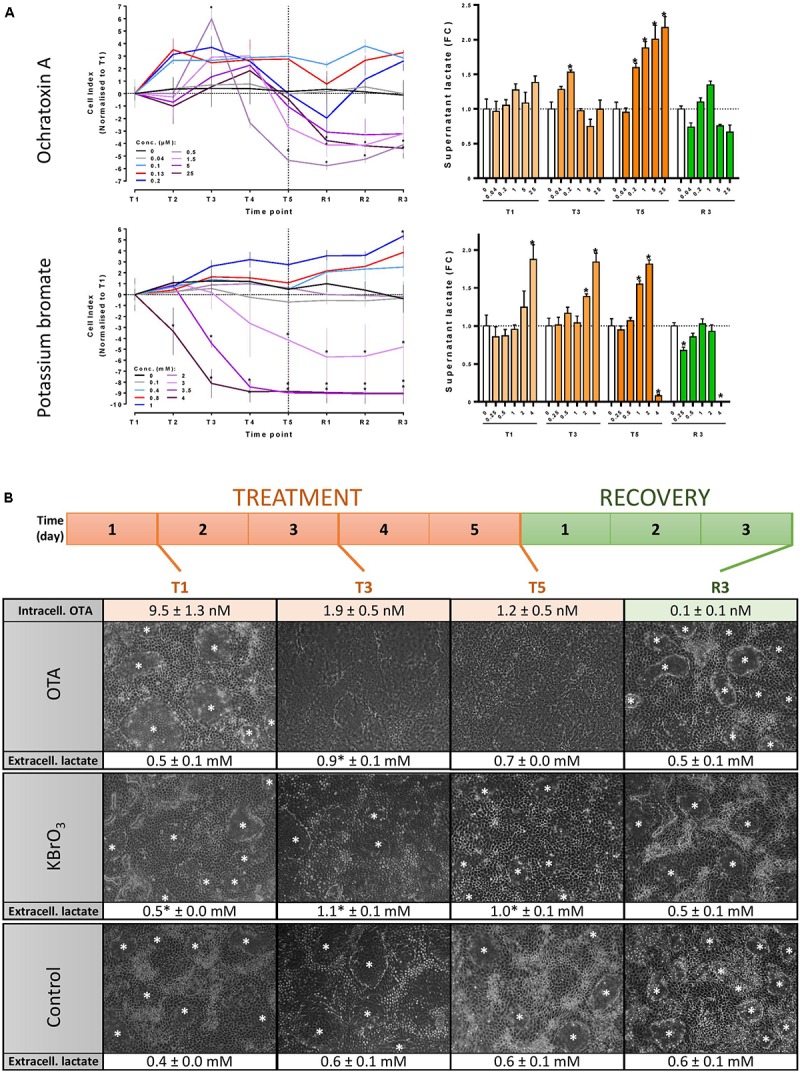
Experimental design optimisation. **(A)** Concentration range testing in 96-well plates: Mature RPTEC/TERT1 cells were exposed to up to 25 μM OTA, 4 mM KBrO_3_ or a vehicle control for 5 days (T5) and fed a further 3 days with medium (R3). Cell viability was assessed by measuring impedance and calculating cell index (CI) with the xCELLigence device. Cellular stress was assessed by measuring supernatant lactate. Significance of change compared to time-matched control in a minimum of 3 biological replicates was assessed with a two-way ANOVA followed by a Bonferroni posttest. ^∗^*p* ≤ 0.05. T1, T2, T3, T4, T5: days 1, 2, 3, 4, and 5 of treatment, respectively. R1, R2, R3: days 1, 2, and 3 of recovery post-treatment. **(B)** Experimental design for the omic study. Mature RPTEC/TERT1 cells monolayers were treated every 24 h for 5 days with 130 nM OTA, 0.8 mM KBrO_3_ or a vehicle control. The treatment was followed by a 3-day recovery period with 24h-feeding cycles with cell culture medium for all three conditions. Cells were lysed for omic investigations at the 4 indicated time points. Intracellular OTA levels were measured by UPLC-MS at the 4 time points. Cell viability and cell stress were monitored throughout the experiment by microscopic examination (original magnification 100×) and lactate measurements in cell culture supernatants. Lactate levels in treated cells were compared to time-matched controls with a two-tailed unpaired Student’s *t*-test (^∗^*p* ≤ 0.05, *n* = 3). White asterisks (^∗^) show domes indicative of vectorial transport.

Test concentrations were 130 nM OTA and 0.8 mM KBrO_3_ for all omic experiments. These concentrations were sub-cytotoxic, but induced cellular stress, as shown in preparatory experiments (Figure 1A). Impedance was measured in the xCELLigence device from ACEA. Cells were seeded onto E-plates in 60 μL medium and differentiated. Impedance reflects the attachment of the cells to the growth support and can therefore be used as a cell viability endpoint in contact-inhibited cell monolayers. Cell index (CI) was measured every 24h and normalized for each treatment condition to the average T1 value (*n* = 3). Decreased CI corresponds to a decrease in cell viability (Limonciel et al., 2018). Increased CI can be seen as a marker of cellular stress, possibly linked to the collapse of dome structures on solid plastic support. Supernatant lactate was quantified using a biochemical assay (Limonciel et al., 2011). Statistical analysis was performed using a two-way ANOVA with a Bonferroni multiple comparisons posttest using GraphPad Prism v6.01 for each dataset (^∗^*p* < 0.05).

### Cell Treatment

Treatments were applied in a bi-phasic regime where the cells were exposed to either OTA, KBrO_3_ or HDM (vehicle control) for 5 days and allowed a 3-day recovery period post-treatment where all cells were exposed to HDM. For both the treatment and recovery phases, cell culture medium was renewed every 24 h. Cell lysates were prepared for omic investigations and OTA quantification after 1, 3 and 5 days of treatment (T1, T3 and T5, respectively) and at the end of the recovery period (R3, day 8 of experiment) (Figure 1B). Supernatant medium was collected for OTA quantification at the same time points. All omic endpoints were measured in three biological replicates.

### RNA Preparation

At each lysis time point, medium was removed and cells were harvested in Qiazol (Qiagen). Total RNA was isolated using a miRNeasy Mini Kit (Qiagen, 217004) according to the manufacturer’s protocol and followed by DNase I (Qiagen) treatment. Upon purification, RNA concentrations were measured with a NanoDrop^®^ ND-1000 spectrophotometer (Thermo Scientific) at 260 and 280 nm. RNA quality and integrity were assessed by automated gel electrophoresis on an Agilent 2100 Bioanalyzer system (Agilent Technologies). Only RNA samples which showed clear 18S and 28S peaks and with an RNA integrity number (RIN) higher than 8 were used. Samples were stored at −80°C until RNA hybridization.

### mRNA Microarrays

The DNA array platform used was the Affymetrix Human Genome U133 plus 2.0 array. CEL files were loaded into BRB array^1^ and normalized using the RMA method. Differences in gene expression measurements under any condition compared to time-matched control were summarized for each probe as log2 fold change (LFC) value. The associated *p*-value was computed using the moderated (unpaired) *t*-test (Smyth, 2004) and was corrected for multiple testing by the Benjamini-Hochberg procedure (Benjamini and Hochberg, 1995). Calculations were performed using the R package “limma” (Smyth, 2005). All transcriptomic data was deposited at Array Express under the accession number E-MTAB-7048.

### miRNA Microarrays

Profiling of miRNA expression was performed using Agilent Sureprint G3 Unrestricted Human miRNA V19 8 × 60 K microarrays. Hybridization was performed following standard protocols, after which the microarray slides were washed and scanned using a DNA microarray scanner (Agilent Technologies). The scanned images were converted into TXT files using the Feature Extraction Software v10.7.3.1 from Agilent Technologies, which were imported in R 2.15.3^2^ for quality control with an in-house developed pipeline (Coonen et al., 2015). Filtering and normalization was performed using AgiMicroRna (López-Romero, 2011). Total gene signals were log2-transformed and quantile-normalized. Differentially expressed microRNAs with an FDR adjusted *p*-value < 0.05 were considered statistically significant.

### DNA Methylation

Cells were washed with HBSS, trypsinised and lysed with a digestion buffer containing 1 mM EDTA, 50 mM Tris–HCl, 5% SDS and 1 mg/mL proteinase K. DNA was extracted and processed for methylated DNA immunoprecipitation (MeDIP) before hybridisation onto Human 2.1 M Deluxe Promoter arrays (Roche NimbleGen, Basel, Switzerland). Detailed procedures are available in the Supplementary Material.

Signal intensity was extracted from images using NimbleScan v2.6 and differentially methylated regions (DMRs) compared to control were identified following analysis with a probe sliding window ANOVA algorithm (sliding window of 750 bp comprising 7 probes and a FDR corrected *p*-value < 0.01). Detail of the analysis can be found in the Supplementary Material and (van Breda et al., 2014). Log2 ratios > 0 indicate hypermethylation and log2 ratios < 0 indicate hypomethylation.

### Histone Acetylation Analyses Using Chip-on-Chip

Chromatin immunoprecipitation was performed using the SimpleChIP^®^ Enzymatic Chromatin IP Kit (Magnetic Beads) (Cell Signaling Technology) as detailed in the Supplementary Material. H3K9 acetylation of human promoters was studied using the Human 2.1 M Deluxe Promoter Array (Roche NimbleGen, Basel, Switzerland). Labelling, hybridization and washing of arrays was performed according to the manufacturer’s protocol as described in the DNA methylation section. Data analysis and selection of differentially acetylated genes was performed with the same workflow as for the MeDiP-chip data.

### Stress Response Pathway Analysis

Pathway genes were chosen based on the lists of target genes previously generated in our group (Limonciel et al., 2015). Genes were ranked based on their average log2 fold over control (LFC) across the three time points of treatment with KBrO_3_, the positive control for Nrf2 and p53 activation (Limonciel et al., 2012). Genes showing no significant modulation by KBrO_3_ were removed from the original list. The remaining genes are ranked based on the average LFC during treatment with OTA.

### GC-MS Metabolomics

For metabolomics, the cells were lysed in ice-cold methanol (MeOH). Cell lysate samples were derivatised for GC-MS by a two-step methoximation/silylation derivatization procedure (Kind et al., 2009). The following derivatization standards were added to the samples: ^13^C-Serine (20 μL, 1 mM), U-^13^C-Glucose (20 μL, 1 mM) and myristic acid d27 (10 μL, 1.5 mg/mL). The dried samples were first methoximated with a solution of 20 mg/mL methoxyamine hydrochloride in anhydrous pyridine (20 μL) and incubated at 30°C for 90 min. Samples were then silylated by adding 80 μL MSTFA (with 1% TMCS) (Thermo) and incubating at 37°C for 30 min. Following derivatization, 2-fluorobiphenyl in anhydrous pyridine (10 μL, 1 mM) was added as an injection standard and the samples were transferred to deactivated glass vial inserts. GC-MS analysis was performed on an Agilent 7890 GC equipped with a 30 m DB-5MS capillary column with a 10 m Duraguard column connected to an Agilent 5975 MSD operating under electron impact (EI) ionization (Agilent Technologies UK Ltd.). Samples were injected with an Agilent 7693 autosampler injector into deactivated splitless liners according to the method of Fiehn and colleagues (Kind et al., 2009) using helium as the carrier gas. One sample was used as a quality control (QC) and injected repeatedly throughout the run to monitor system performance. Metabolites were assigned using the Fiehn Library with the deconvolution program AMDIS (Stein, 1999), and Matlab program GAVIN, developed in-house, was used to integrate metabolite peak areas for all samples (Behrends et al., 2011). Data was normalized by the QC-RLSC method described by Dunn et al. (2011). Statistical significance of the change induced by OTA or KBrO_3_ was assessed using a two-way ANOVA with a Sidak posttest in GraphPad Prism v6.05. Significant changes (*p* ≤ 0.05) are indicated in bold in Figure 7.

### OTA Quantitation by UPLC-MS

For intracellular extracts, 150 μL aliquots of the MeOH extracts were dried under reduced pressure in a speedvac, and resuspended in 1:9 acetonitrile:water (100 μL) using UPLC grade solvents (Romil LTD, Code H949, Cambridge, United Kingdom). Sample solutions were then transferred to high recovery chromatography vials (Waters Corporation, Milford, MA, United States). For cell culture medium samples, 100 μL aliquots of the media were added to 300 μL MeOH. Samples were vortexed and then centrifuged at 16000 *g* for 5 min. Supernatants were transferred to high recovery chromatography vials and concentrated under reduced pressure in a speedvac, before resuspension in 1:9 acetonitrile:water (100 μL). Standard solutions of OTA ranging from 100 to 0.001 ng/mL (248 to 0.002 nM) were prepared in 1:9 acetonitrile:water (100 μL) and transferred to high recovery chromatography vials. Reversed-phase chromatographic separation of the cell lysates was conducted using an Acquity UPLC system (Waters Corporation, Milford, MA, United States) on an Acquity HSS T3 C18 column 10 mm × 2.1 mm, 1.8 um (Waters) and a binary gradient elution comprising water +0.1% formic acid (Sigma) and acetonitrile +0.1% formic acid, with an injection volume of 15 μL. Mass spectrometric analysis of the chromatographic eluent was performed using a quadrupole time-of-flight (QtoF-Ultima) spectrometer (Waters) with data collected in centroid mode in the m/z range 70–1000. Analysis was performed in positive ion mode electrospray ionization. The elution gradient was as follows: 99.5% A at 0 min-99.5% at 3 min to 99.5% B at 19 min- 99.5% B at 23 min, 99.5% A at 23.1 min, 99.5% A at 27 min. The column was kept at 50°C and the auto-sampler at 4°C. Limit of detection (LOD) was 0.12 nM and limit of quantitation (LOQ) was 0.32 nM.

### Statistical Analysis

For all omics and OTA quantification, three biological replicates were produced. Statistical analysis is reported in the respective section for each method. For correlation of epigenetic modifications with gene expression levels (Figure 3A), the correlation of differentially expressed genes with epigenetic modifications was tested with Spearman’s rank correlation coefficient (ρ) at each time point. Strong correlation of HA or DM with the direction of gene expression changes renders a coefficient close to 1, strong anti-correlation is represented by a coefficient close to −1. A *p*-value for statistical significance of the association was also calculated and is reported by asterisks when significant (^∗^*p* < 0.05, ^∗∗^*p* < 0.01 and ^∗∗∗^*p* < 0.001).

## Results

### Concentration Range Testing and OTA Uptake

The concentrations of OTA and KBrO_3_ used for the omic investigations were chosen after rigorous concentration range testing in RPTEC/TERT1 cells to cause a minimal decrease in cell viability (impedance/CI) and an increase in cellular stress (extracellular lactate) (Figure 1A). Based on these results, three biological replicates of differentiated RPTEC/TERT1 cells were exposed to 130 nM OTA, 0.8 mM KBrO_3_ or a vehicle control in a 5-day repeat-dose exposure regime followed by a 3-day recovery period with compound washout and cell culture medium renewal every 24 h (Figure 1B). There was no significant cell death throughout the omic experiment with either chemical. However, both caused an increase in the stress marker lactate during treatment. OTA also caused the disappearance of dome structures indicative of vectorial transport of water and solutes in proximal tubule cells cultured on solid support (Wilmes et al., 2014). The cells were lysed for transcriptomic, epigenomic (histone acetylation and DNA methylation arrays), miRNA and metabolomic investigations at 4 time points: after 1, 3 and 5 days of treatment (T1, T3 and T5, respectively) and after 3 days of recovery post-treatment (R3).

Ochratoxin A itself was quantified in cell lysates at the 4 time points by UPLC-MS, revealing that after 24 h of exposure, 9.5 ± 1.3 nM OTA were present in the cells (Figure 1B). Intracellular levels were lower in the following days, demonstrating a lack of accumulation of the parent compound in spite of 5 consecutive exposures between T1 and T5. In the supernatant OTA remained close to 90 nM at all treatment time points and was below 0.3 nM (LOQ) at R3 (data not shown). At R3, cellular OTA was negligible (less than 0.12 nM), demonstrating an effective washout of the chemical. Interestingly, a product of hydrolysis of OTA, OTAα, was also detected with a peak at T3. Low intracellular levels of OTAα were still detectable after recovery. Taken together, these data suggest that the nominal concentration is close to the actual treatment concentration, that OTA enters the cell, where it is hydrolysed to OTAα and that a 3-day washout effectively reduces the internal concentration of OTA and its metabolite.

### Quantitative Impact on Gene Expression and Regulatory Mechanisms

**FIGURE 2:**
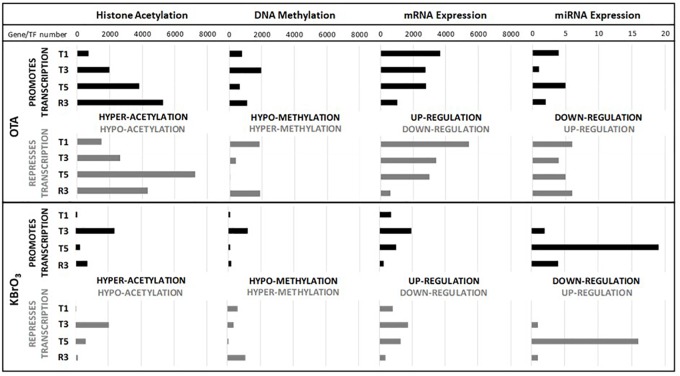
Quantitative impact on gene expression and epigenetic modifications. The number of hypo- and hyper- acetylated histones/methylated genes was based on the sign of the difference of the median treatment and the median control values. For mRNA, the probe list was reduced per time point and treatment with a cut off on *p*-value of change of 0.001. When several probes for a given gene remained, the probe with the highest variation in the condition was chosen. No cut off on the intensity of change was applied. For miRNA, the probe list was reduced per time point and treatment with a *p*-value cut off of 0.05. For each endpoint, the number of changes is represented on the *X* axis and the different time points (T1, T3, T5, R3) on the *Y* axis.

The epigenomic and transcriptomic datasets revealed a very large impact of OTA on histone acetylation (HA) and mRNA expression and a milder effect on DNA methylation (DM) and miRNA expression (Figure 2). At T5, OTA had induced significant changes compared to time-matched controls on 11047 genes for HA, 5793 genes for mRNA, 639 genes for DM and 10 miRNAs. Interestingly, the largest impact on mRNA expression occurred after the first 24h of exposure (9102 genes), likely through a direct impact on transcription or mRNA processing, while the impact on HA was strongest on the last day of treatment (T5) and after recovery (R3). In comparison, KBrO_3_ had a much smaller quantitative impact on epigenetics and mRNA expression, but modulated more miRNAs than OTA, especially at T5 (Figure 2).

**FIGURE 3:**
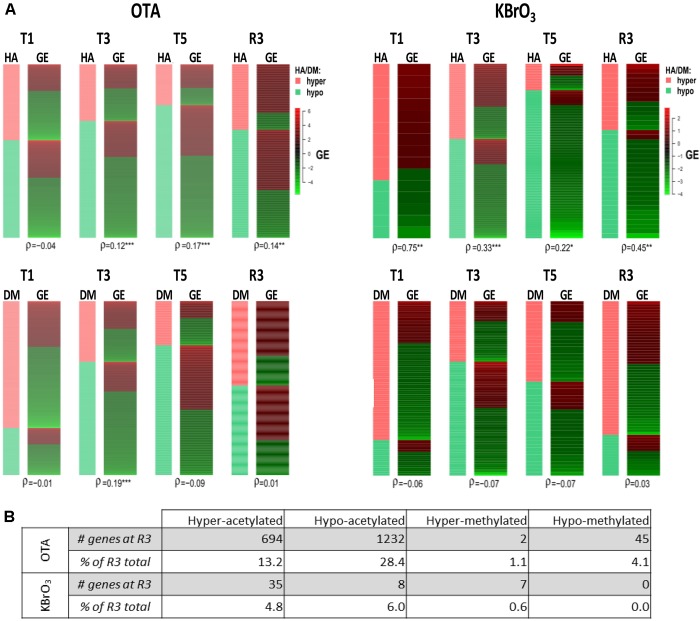
Correlation of epigenetic modifications to mRNA expression changes and persistence after washout and recovery in OTA- and KBrO_3_-treated cells. **(A)** - Correlation between epigenetics and gene expression was tested at each time point with Spearman’s rank correlation coefficient (ρ). Strong correlation between histone acetylation (HA) or DNA methylation (DM) and mRNA gene expression (GE) renders a coefficient close to 1, strong anti-correlation renders a coefficient close to −1. A *p*-value for statistical significance of the correlation was also calculated and is indicated by ^∗^*p* < 0.05, ^∗∗^*p* < 0.01, ^∗∗∗^*p* < 0.001. **(B)** Persisting epigenetic modifications after compound washout and recovery period. Here, epigenetic modifications with sustained directionality at T5 and R3 for a given gene were considered persisting after treatment. Percentage of persistent modifications was calculated as the percentage of modifications in the R3 datasets that were already present at T5 and modified in the same direction compared to time-matched control; e.g., for OTA, 694 genes corresponding to 13.2% of hyper-acetylated genes at R3 were already hyper-acetylated at T5.

A global analysis of the correlation of HA/DM status with modulated gene expression (GE) at each time point for both compounds was conducted (Figure 3A). The results show that the strongest correlation was between HA and GE in KBrO_3_-treated cells with a maximum at T1 (correlation coefficient ρ = 0.75). Although OTA had a dramatically stronger effect on both HA and GE in terms of number of impacted genes, the correlation between HA and GE in OTA-treated cells was much weaker, suggesting a weak contribution of HA to GE modulation in spite of a very high number of differentially acetylated histones. For DM, where anti-correlation with GE would be expected, neither compound showed a strong anti-correlation.

**FIGURE 4:**
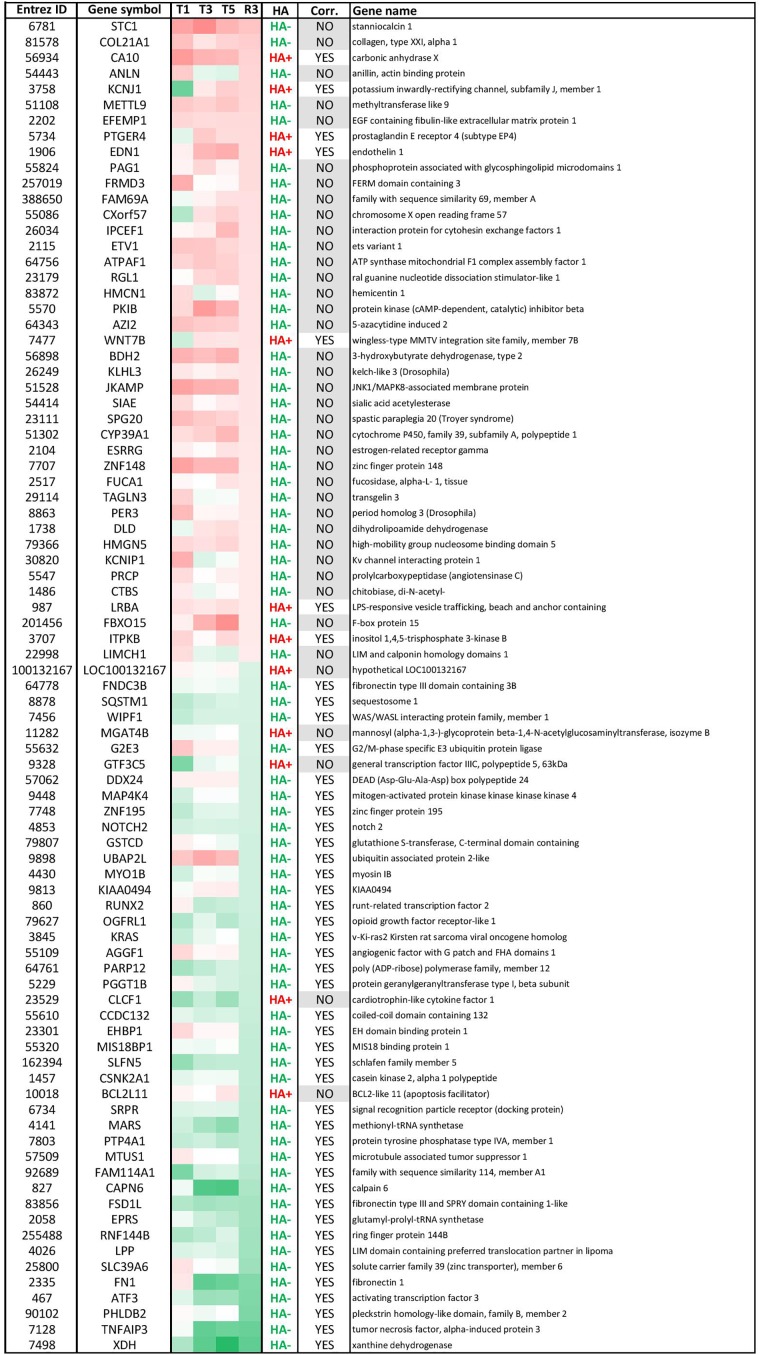
Modulated genes with persistent histone acetylation (HA) modification after OTA washout. These 85 genes had (1) significantly altered mRNA levels at R3 (± 0.58 LFC) and (2) persistent HA modifications from T5 to R3. Within this list, 44 genes had HA modification consistent with the direction of GE (correlation: YES, i.e., up-regulated GE/HA+ or down-regulated GE/HA-) and 41 had inconsistent HA modifications (correlation: NO). Most genes had hypo-acetylated histones. Red corresponds to up-regulated GE. Green corresponds to down-regulated GE.

Effects of OTA on HA regulation have already been reported, notably through inhibition of HATs and activation of HDACs, which would favor a global decrease in histone and overall protein acetylation (Marin-Kuan et al., 2006). While in this study HA was strongly impacted in presence of OTA and after compound washout, Figure 3B shows that the majority of epigenetic modifications observed at R3 were new and not conserved from treatment. The most conserved modifications were hypoacetylations with 28% of R3 hypoacetylations already present at T5. However, when cross-analyzing persistent hypoacetylations with significantly modulated mRNAs in the OTA dataset (set of 85 genes-Figure 4), 44 genes showed a directionality of gene expression consistent with histone modifications, vs. 41 genes with histone modifications that would favor the opposite effect on gene expression. In the KBrO_3_ dataset, 43 genes had persistently differentially acetylated histones from T5 to R3. Only 3 of those genes were also differentially expressed at R3 compared to control: CCL2, TGFB2 and SRRM2. All three genes were down-regulated at R3, CCL2 and TGFB2 were hypo-acetylated and SRRM2 was hyper-acetylated. Thus, even with a focus on persisting histone modifications, there was no global correlation between HA and GE with either chemical, suggesting a marginal effect of HA modulation *alone* on mRNA expression on a global scale.

**FIGURE 5:**
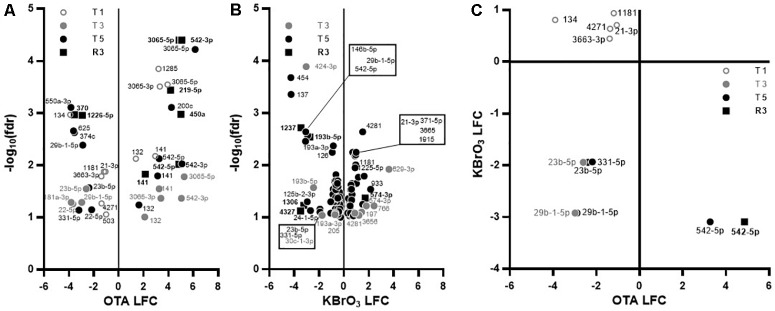
Effects on miRNA expression in RPTEC/TERT1 cells. **(A,B):** Differentially expressed miRNAs in OTA- **(A)** and KBrO_3_- **(B)** treated cells. Significance of change compared to time-matched control was set to *p* ≤ 0.1 based on false discovery rate (fdr) and plotted as log_2_ fold change (LFC) vs –log_10_(fdr). There was no significant change at T1 in KBrO_3_-treated cells. **(C):** Relative expression of miRNAs significantly impacted by KBrO_3_ at T5 and by OTA at the indicated time point, regardless of the direction. Changes occurring with one chemical only are not represented.

In the miRNA dataset, while KBrO_3_ induced the most deregulations at T5, very few miRNAs overlapped with any other condition, suggesting a peak of miRNA production that is absent in the OTA dataset. In contrast, while only a maximum of 10 miRNAs were affected at any time point with OTA, most of the up-regulated miRNAs were impacted at several time points, with four of them still up-regulated at R3: miR-3065-3p, miR-141, miR-542-3p and miR-542-5p. In contrast, miR-450a and miR-219-5p were up-regulated and miR-1226^∗^ and miR-370 were down-regulated in recovery exclusively (Figure 5A). These last two miRNAs were also down-regulated in the KBrO_3_ dataset, after recovery only (Figures 5B,C). miR-132 was heavily induced by OTA treatment only. Figure 5C compares the changes in miRNAs in both treatments based on log2 fold change (LFC). Two miRNAs (miR-23b, miR-29b-1) were similarly down-regulated by both treatments after repeated exposures (T3 and T5). Five miRNAs were down-regulated by OTA but up-regulated by KBrO_3_ treatment at T1 (miR-21-3p, miR-1181, miR-134, miR-3663-3p and miR-4271). Interestingly, the two chemicals had opposite effects on miR-542-5p expression, both at T5 and R3, where its levels were increased by OTA and decreased by KBrO_3_.

### Impact on Stress Response Pathways

**FIGURE 6:**
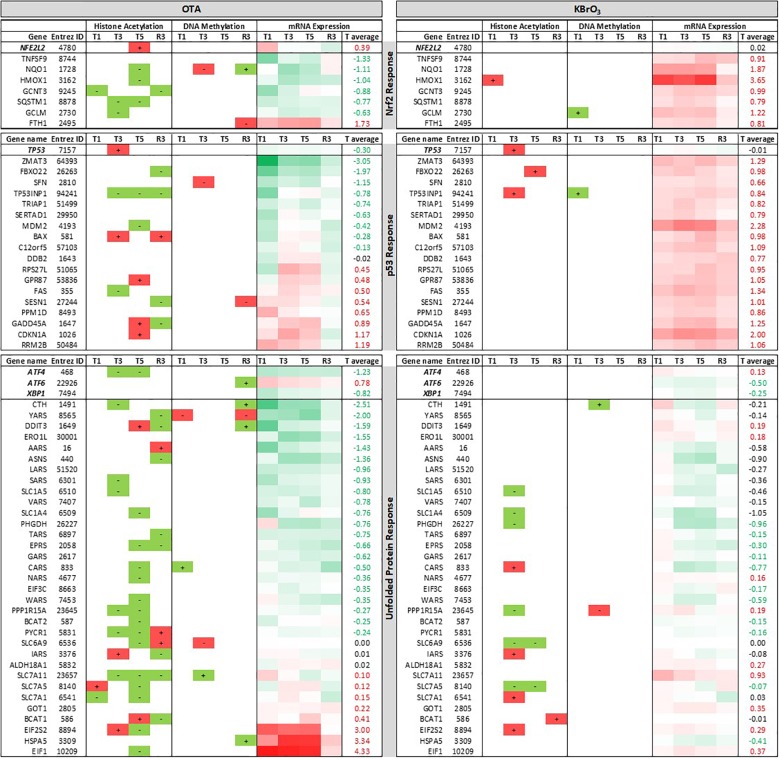
Epigenetic and mRNA changes associated with stress response pathways. Three stress response pathways are described: the Nrf2 response to oxidative and alkylating stress, the p53 response to DNA damage and the unfolded protein response. Gene lists include the governing transcription factors Nrf2 (NFE2L2), p53 (TP53), ATF4, ATF6 and XBP1, followed by some of their target genes. For HA and DM, “+” means hyper- and “–“ means hypo-modifications compared to time-matched control (in all 3 replicates). Red symbolizes modifications that classically promote gene expression for HA and DM / mRNA up-regulation. Green symbolizes modifications that classically reduce gene expression for HA and DM / mRNA down-regulation. Heatmap for mRNA expression ranges from 4 to −2 and is centered to 0 (white). “T average” is the average log2 fold change compared to time-matched control. For each pathway, genes are sorted in descending order of T average in the OTA dataset.

The impact of both chemicals on the expression on Nrf2, p53 and unfolded protein response (UPR) related genes, as well as the epigenetic modifications identified on the transcription factors and their target genes are shown in Figure 6. KBrO_3_ induced the up-regulation of Nrf2 and p53 target genes, yet with very few corresponding HA and DM modifications. In contrast, KBrO_3_ did not alter the expression of the UPR targets studied here.

In OTA-treated cells, several Nrf2 and p53 targets were down-regulated during treatment. In line with the large impact of OTA on HA, most of the genes studied in Figure 6 showed HA modifications at at least one time point, however there was no sign of a consistent epigenetic modulation with a lasting effect on gene expression. Only two miRNAs impacted by OTA had validated targets from this list: miR-1285-3p targeting TP53 and miR-132-3p targeting CDKN1A (p21). The transcripts of two of the UPR-driving transcription factors (ATF4 and XBP1) were down-regulated in OTA-treated cells. Several of their target genes were also down-regulated, with the notable exception of HSPA5 (up-regulated from T1 to T5), which encodes the protein BiP responsible for protein misfolding sensing in the ER and the activation of all three branches of the UPR. Two elongation factors (EIF2S2 and EIF1), involved in translation, were also consistently up-regulated during OTA treatment and recovered after washout.

Altogether, these results suggest an inhibition of the Nrf2, p53 and unfolded protein responses by OTA at gene expression level, which does not appear to be driven primarily by epigenetic mechanisms (HA, DM or miRNA).

### Metabolic Impact

#### Amino Acids

**FIGURE 7:**
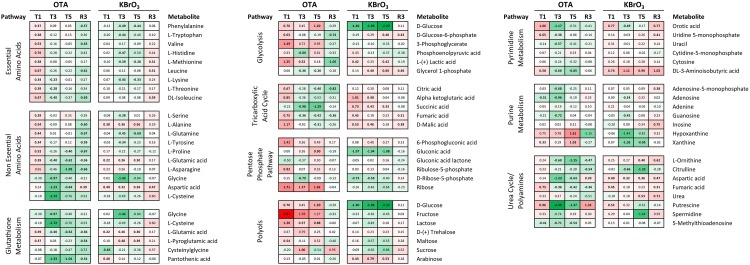
Effects on metabolite levels in RPTEC/TERT1 cell lysates. Intracellular metabolites were measured in methanol lysates by GC-MS at the four indicated time points. Data is shown as log2 fold over time-matched control (LFC). Metabolites are grouped per pathway and appear multiple times if shared between pathways. Heatmap ranges from 3.62 (fructose / OTA T1, red) to –2.22 (glucose / KBrO_3_ T5) and is centered to 0 (white). Bold LFCs indicate LFC significantly different from control, as determined by 2-way ANOVA with Sidak posttest (*p* ≤ 0.05).

Metabolomic analysis of the intracellular contents of OTA-treated cells revealed an increase in both essential (histidine, isoleucine, leucine, valine, threonine, methionine, tryptophan phenylalanine and lysine) and non-essential (alanine, proline, glutamine, glutamic acid, serine, tyrosine) amino acids after the first 24 h of treatment, with the notable exception of the non-essential amino acids aspartic acid, cysteine and glycine that are all important in the synthesis of the antioxidant glutathione (Figure 7). This early response was not sustained at later treatment time points, where most amino acid deregulations were toward a depletion, with the most striking effects on aspartic acid, cysteine and glycine at T3. After OTA washout and recovery (R3), the depletion of several essential (isoleucine, leucine, valine, phenylalanine) and non-essential (asparagine, alanine, proline, glutamine, glutamic acid, serine, tyrosine) amino acids could still be measured, while aspartic acid levels were increased compared to time-matched controls.

Upon exposure to KBrO_3_, the metabolomic profile was very different, with a mild impact only on non-essential amino acids at T1, but which was sustained until T5 (increased levels of alanine, glutamic acid, aspartic acid). In addition, serine and tyrosine levels were decreased at T3, but returned to control level at T5, while glutamine was depleted at both time points. Essential amino acids were depleted at the late treatment time points only: histidine, methionine, tryptophan, phenylalanine and lysine at T3; methionine, phenylalanine and lysine at T3 and T5. After KBrO_3_ washout and recovery, amino acid levels were still not back to control levels. In particular, isoleucine, leucine, methionine and aspartic acid levels were still elevated compared to time-matched control (Figure 7).

#### Metabolic Pathways

Ochratoxin A also largely affected metabolites related to cellular energy production and nucleotide biosynthesis and degradation (Figure 7). Several intermediates of glycolysis and the TCA cycle were significantly impacted by OTA at T1, with glucose, glucose-6-phosphate, 3-phosphoglycerate, lactic acid, citric acid, alpha ketoglutaric acid, fumaric acid and malic acid levels all increased after 24 h. At later exposure time points, succinic acid (decreased) was the only modulated metabolite of these two pathways. After OTA washout and recovery, the first (glucose-6-phosphate) and last (lactic acid) metabolites of glucose degradation through glycolysis were decreased, as well as citric acid. Interestingly, fructose (among the polyols) was consistently increased throughout OTA exposure (strongest increase in the metabolomic dataset) but not at R3. KBrO_3_ did not affect fructose levels, but consistently caused a depletion in glucose in the cells, which was recovered at R3. In addition, KBrO_3_ induced an increase at T1 in all TCA intermediates measured except citric acid. These higher levels were sustained until T3 for alpha ketoglutarate and malic acid and until T5 for succinic acid. At R3, fumaric and malic acid levels were still above control levels after KBrO_3_ washout.

Metabolomics also revealed an impact on the pentose phosphate pathway (PPP), which can branch from glycolysis (C6 metabolism) to provide C5 ribose derivatives, notably for nucleotide biosynthesis. OTA caused an increase in 6-phosphogluconic acid (T1), D-ribose (T1, T3, and T5) and ribose-5-phosphate (T3). KBrO_3_ depleted the levels of glucuronic acid from T1 to T5 and of ribose-5-phosphate at T1 and T3 only, without a significant impact on ribose itself. Both chemicals caused an increase at T1 and a decrease at T3 of the intracellular levels of orotic acid, a downstream metabolite involved in the early steps of pyrimidine biosynthesis. An increase was also measured after recovery from KBrO_3_ but not from OTA. Cytosine and CMP levels were not impacted by either chemical, while uracil and UMP levels were affected at the early time points by OTA only. The degradation product 3-aminoisobutyric acid was consistently increased by KBrO_3_ during exposure and after washout, while OTA’s effects followed the pattern of other pyrimidine metabolites with an increase at T1 and a decrease at later treatment time points.

Regarding purine metabolites, OTA caused a depletion in adenosine (T1, T3), AMP (T3), guanosine (T3) and 5′-methylthioadenosine (5′-MTA; T1, T3, T5, also in the polyamine pathway). OTA exposure also resulted in an accumulation of purine degradation products (hypoxanthine and xanthine) at T5, while the levels of both were back to control level after washout. The levels of adenine were not affected by either chemical. KBrO_3_ decreased the levels of adenosine and guanosine at T3 and caused a mild increase in AMP and inosine after washout. Contrary to OTA, KBrO_3_ caused a decrease in xanthine levels at T3 and T5.

A set of metabolites involved in the urea cycle was particularly impacted by OTA treatment, as well as after recovery (Figure 7). OTA decreased the levels of ornithine, aspartic acid and fumaric acid without affecting the levels of urea itself. In addition, the levels of putrescine, a polyamine metabolite, were alternately increased at T1, strongly decreased at T3 and T5 and again increased at R3. KBrO_3_ had a different effect on urea cycle metabolites, affecting primarily citrulline (decreased at T3 and T5) and urea itself (increased at T5 and R3). KBrO_3_ also impacted the levels of putrescine, although to a lower extent. The metabolite 5′-MTA, a product of spermidine and spermine metabolism, was decreased by OTA at all exposure time points and levels were recovered after OTA washout. KBrO_3_ did not impact the levels of 5′-MTA.

## Discussion

Renal proximal tubule cells are the primary target of OTA toxicity, likely due to basolateral organic anion transport at this site (Tsuda et al., 1999). Previous studies have shown that OTA induces a severe alteration of gene expression *in vitro* in proximal tubule cell models and *in vivo* in the rat renal cortex (Jennings et al., 2012). However, despite a strong impact on the transcriptome, the common toxicologically relevant pathways were not directly impacted with exception of an unusual suppression of the Nrf2 pathway (Limonciel and Jennings, 2013). Thus, transcriptomics alone, particularly in single dose applications, does not reveal a clear mechanism of OTA induced nephrotoxicity and/or carcinogenicity. Here we investigated molecular perturbations induced by OTA at the epigenetic and metabolic levels in repeat dose exposures and in recovery experiments. The effects of OTA were compared and contrasted to those induced by KBrO_3_, a well described nephrotoxin and renal carcinogen with a firm mechanism of toxicity based on oxidative stress and genotoxicity (Limonciel et al., 2012).

Ochratoxin A exhibited cytotoxicity in repeat dose exposures at 5 μM and above at T5 and was even more cytotoxic at these concentrations after recovery (R3). Cellular stress, as measured by increased lactate production (Limonciel et al., 2011), occurred at T3 at the chosen concentration of 130 nM. This concentration, while non-cytotoxic, also inhibited dome formation, an indicator of vectorial transport of water and solutes in proximal tubule cells grown on solid support (Wilmes et al., 2014) by T3. Transport function fully recovered at R3, as evidenced by the reappearance of domes. Measurement of OTA concentrations within the cells, showed a peak of the parent compound at T1, decreasing at T3 and T5. Approx. 1 % of the T1 peak was detected at R3. It is likely that OTA is metabolized quickly to OTAα, which peaked intracellularly at T3 and was only slightly above the limit of detection after recovery. For comparison, a 0.8 mM non-cytotoxic concentration of KBrO_3_ was chosen for the omic studies. This concentration exhibited similar effects on the cells, including elevated supernatant lactate and mildly decreased transport capacity at T3, with full apparent recovery at R3.

Ochratoxin A at 130 nM exhibited a more severe effect on gene expression, histone acetylation and DNA methylation than KBrO_3_. While gene expression somewhat recovered after removal of OTA, both histone hyper-acetylation and DNA hyper-methylation peaked. For both compounds, there was a correlation of histone acetylation to gene expression, however, the correlation was much weaker for OTA. The strongest correlation of the data set was KBrO_3_ at T1, which gradually decreased with repeated dose and increased again in recovery. The opposite was true for OTA, with a poor correlation at T1 that increased at T3 and T5. This analysis suggests that histone acetylation is not the driving force for OTA-induced gene transcription. This disassociation of histone acetylation and gene expression may point to a mechanism of toxicity of OTA. Regarding DNA methylation, neither compound showed a consistent anti-correlation with gene expression.

Ochratoxin A exposure exhibited a minor effect on miRNA expression, affecting 10 miRNAs altogether, whereas KBrO_3_ exposure resulted in a differential expression of 35 miRNAs at T5. A possible explanation for OTA’s imbalance in mRNA / miRNA alterations is an inhibition of the miRNA maturation machinery, as previously reported (Dai et al., 2014; Zhao et al., 2017). Previous reports on the effect of OTA on miRNA expression in HEK293 and HepG2 cells (Zhao et al., 2017) and in GC-2 cells (Chen et al., 2015) have no overlaps with our study. However, the study by Hennemeier et al. (2014) demonstrates the implication of miR-29b (down-regulated by both OTA and KBrO_3_ in our study) in collagen formation in HEK293 cells. In addition, in our study OTA induced the expression of several miRNAs at all treatment time points including miR-3065-5p, miR-141, miR-542-5p, miR-542-3p and miR-132. miR-132 is of potential mechanistic interest as it has previously been shown to be involved in the suppression of Nrf2 genes, with a miR-132 antigomir being capable of preventing OTA-induced Nrf2 mRNA depletion in LLC-PK1 cells (Stachurska et al., 2013).

Indeed, analysis of the OTA-induced transcriptome alterations demonstrated that Nrf2 target genes HMOX1, NQO1, GCLM, TXNRD1 and SRXN1 were severely attenuated whereas all were robustly induced by the oxidant and Nrf2 activator KBrO_3_. It has been well-documented that OTA can induce reactive oxygen species (ROS) (Schaaf et al., 2002; Costa et al., 2016). Thus, it is counter-intuitive that the Nrf2 response that protects the cell against oxidative stress be attenuated by a ROS-inducing chemical. However, this is a striking finding in this study and has been reported by us and several other groups (Limonciel and Jennings, 2013). The mechanism of OTA-induced Nrf2 response inhibition is not clear and is potentially based on inhibition of Nrf2 translocation, induction of miR-132, inhibition of protein acetylation through HDAC activation and HAT inhibition or combinations of all. In any case, it is plausible that OTA-induced Nrf2 inhibition renders the cell defenseless to oxidative injury, potentially leading to increased cell death rates and cancer.

Within the UPR pathway, OTA induced ATF6 and HSPA5 (aka BiP) transcription, but suppressed ATF4 and many of its target genes, including tRNA synthetases (YARS, AARS, LARS, SARS, VARS, TARS, EPRS, GARS, CARS, NARS, WARS, IARS) and amino acid transporters (SLC1A5, SLC1A4, SLC6A9, SLC7A11). All of these may point to a general increase in protein turnover. Indeed OTA’s strong affinity for serum albumin is responsible for its high plasma half-life of up to 35 days (Studer-Rohr et al., 2000). It is conceivable that OTA also binds to cytosolic and cytoskeletal proteins with high affinity initiating proteasomal degradation and autophagy.

We have previously demonstrated an interaction of the Nrf2 and ATF4 pathways in the maintenance of glutathione levels after oxidative injury (Wilmes et al., 2013). Since Nrf2 also induces mRNA expression of ATF4, it is possible that inhibition of the Nrf2 pathway also suppresses the ATF4 branch of the UPR. In the UPR, ATF4 primarily orchestrates the expression of amino acid transporters and aminoacyl-tRNA synthetase enzymes that attach amino acids to their specific tRNAs for inclusion in newly translated proteins (Jennings et al., 2013). Metabolomic analysis showed an OTA-induced increase in all essential amino acids at T1, which could result from an abrupt interruption of translation at the beginning of exposure or an increase in amino acid transport from the cell culture medium that was not sustained at later time points. For non-essential amino acids, however, while most metabolites were increased at T1, many were strongly decreased at all the other time points, including R3. In particular at T3, the glutathione building blocks glycine and cysteine were decreased, suggesting an impact on the capacity of the cells for *de novo* glutathione synthesis.

KBrO_3_ exhibited a strong induction of genes in the p53 pathway. OTA caused a weaker response although some p53 genes were robustly induced, including CDKN1A (p21) and GADD45A. The p53 pathway is an important regulator of many processes including DNA damage and glycolysis. Although both chemicals increased lactate production, OTA and KBrO_3_ had very different impacts on other metabolites involved in glucose metabolism through glycolysis (of which lactate is the final metabolite), the tricarboxylic acid (TCA) cycle, the PPP and the polyol pathway. In the latter, glucose is converted to sorbitol by aldo keto reductases (AKR1B1, AKR1B10) and then to fructose by sorbitol dehydrogenase (SORD). In OTA-treated cells, fructose was consistently increased, suggesting an activation of the polyol pathway, as fructose is not present in the cell culture medium used. In addition, SORD, encoding the enzyme that converts sorbitol to fructose, was amongst the strongest up-regulated genes in the OTA gene expression dataset. Its log2 FC was 5.0 at T1, 5.3 at T3, 5.0 at T5 and still 1.0 at R3 (2 folds above time-matched control).

Another interesting aspect of the metabolomic dataset was the impact on nucleotide metabolism that could interfere with the availability of nucleotides for mRNA synthesis or reflect an attempt at *de novo* nucleotide biosynthesis, possibly to support DNA repair mechanisms.

The polyamine metabolism pathway was also particularly affected by OTA. Putrescine, a metabolite of ornithine, was impacted by OTA at all time points. However, putrescine is available from the cell culture medium, thus the increases measured at T1 and R3 could be the result of increased uptake by the cells. The decreases in putrescine levels at T3 and T5, however, suggest an interference with its further degradation to spermidine, of which the levels were not significantly changed by OTA. 5′-MTA, a by-product of spermidine and spermine synthesis, on the other hand was consistently decreased during OTA exposure. 5′-MTA has many roles such as being an inhibitor of polyamine synthesis (Evans et al., 2004), as a starting point for the purine and methionine salvage pathway (Williams-Ashman et al., 1982) and as a methyl donor for methylation of other molecules (Avila et al., 2004). This last property is of particular interest in the context of DNA methylation, as OTA has been shown to cause a global hypomethylation of DNA in HepG2 cells (Zheng et al., 2013).

As a food contaminant of great concern, OTA has been at the center of several studies focusing on the use of metabolomics to identify new biomarkers of exposure in blood and urine. Male rats exposed to up to 210 μg OTA/kg bw by gavage for up to 90 days had elevated glucose, lactate, alanine and glycine levels in the urine, while citrate and oxoglutarate levels were decreased compared to control (Sieber et al., 2009). Another study with similar exposure up to 26 weeks found elevated levels of alanine and threonine in the rats’ blood associated with low blood glucose and high lactate (Xia et al., 2014). This study also found high levels of fumarate, malate (increased at T1 in our study), ribose (increased throughout treatment), uridine, sorbitol, fructose (increased at T1), aspartic acid (decreased at late treatment time points, increased at R3), leucine, serine, proline (all 3 increased at T1) and ornithine (decreased at late treatment time points and R3) in the biofluids analyzed. A single dose of OTA administered to male rats (6.25 mg OTA) was also shown to cause a modulation in the levels of citrate and an increase in oxoglutarate, lactate, glucose and succinate levels in the urine (Mantle et al., 2011). Although these studies focus on extracellular fluids, our analysis of the intracellular contents shows an overlap for many of the features identified as potential biomarkers of OTA exposure *in vivo*. Metabolomic analysis was previously performed both in cell lysates and supernatants of RPTEC/TERT1 exposed to both chemicals for up to 3 days (1mM KBrO_3_ / 300 nM OTA) (Ellis et al., 2011). After a bolus exposure, extracellular lactate and pyruvate levels were increased (intracellular levels were unchanged), while glucose was depleted from the medium with both chemicals. Intracellular betaine was decreased by both chemicals. In addition, KBrO_3_ caused an increase in alanine, glycerophosphocholine and total glutathione within the cells. Thus the features identified in the Ellis et al. study further support the deep interference with energy metabolism identified for both chemicals in our study.

Taken together these results support previous reports of the effect of OTA on metabolic processes related to protein synthesis (amino acid availability), nucleotide synthesis and energy metabolism, as well as the effect of both chemicals on stress responses to oxidative stress and DNA damage (activated by KBrO_3_, inhibited by OTA). The exhaustive metabolomic investigation is concordant with previous reports of the effects of OTA and brings further insights on the effects of KBrO_3_ on the metabolome. While the large effect of OTA on gene expression and epigenomic regulation had been previously reported, we show here that the effects on histone acetylation and DNA methylation, do not appear to be a driving force in the large transcriptional impact of OTA in renal proximal tubule cells. It is likely that OTA uptake into the cell initiates several simultaneous perturbations including proteotoxicity, disturbances of the histone machinery, Nrf2 inhibition, DNA injury and perturbations of glucose catabolism. Further work will be needed to delineate these mechanisms and to uncouple which mechanisms are direct OTA effects and which are compensatory mechanisms.

## Author Contributions

The experiments were designed by AL and PJ. The cell culture experiments were conducted by AL, AW, and LA. miRNA and epigenomic samples analysis was coordinated by SvB, TdK, and JK. Metabolomic samples analysis was coordinated by GT, APS, and HK. mRNA samples measurement was coordinated by AS. Transcriptomic data normalization and correlation to epigenomics was conducted by XJ and AK-S. Data was analyzed and integrated by AL. Manuscript was primarily written by AL and PJ with contribution from all co-authors.

## Conflict of Interest Statement

The authors declare that the research was conducted in the absence of any commercial or financial relationships that could be construed as a potential conflict of interest.

## Footnotes

1 http://linus.nci.nih.gov/BRB-ArrayTools.html

2 http://www.r-project.org
