# Supplementary material for: Persistence of Epigenomic Effects After Recovery From Repeated Treatment With Two Nephrocarcinogens
Source: Front Genet. 2018 Dec 3;9:558. doi: 10.3389/fgene.2018.00558 (PMC6286959; doi:10.3389/fgene.2018.00558)
Supplement: Supplementary file 1 [file Data_Sheet_1.docx]

Supplementary material

**Persistence of epigenomic effects after recovery from repeated treatment with two nephrocarcinogens (Limonciel et al.)**

**Materials and methods**

***DNA Methylation***

*Sample preparation:* Cells were washed twice with Hanks’ Balanced Salt Solution (HBSS) (Invitrogen), and subsequently trypsinized for 5 min at 37°C. HBSS was added in order to neutralize the trypsin. The cell suspension was centrifuged for 5 min at 500 g to pellet cells. Supernatant was discarded and the cell pellet was lysed in 500 µL digestion buffer (1mM EDTA, 50mM Tris–HCl, 5% SDS, pH 8.0) and 25 µL proteinase K (1mg/mL) (Ambion, Bleiswijk, The Netherlands). After a 1h incubation at 55°C, proteinase K was inactivated for 10 min at 80°C. Next, 2 µL RNase A (100 mg/mL, Qiagen) were added for 1h at 37°C. Organic extraction was performed by adding 1:1 (v/v) phenol-chloroform-isoamylalcohol (25:24:1 v/v) (Sigma-Aldrich, Zwijndrecht, the Netherlands). The mixture was shaken manually for 5 min and centrifuged for 5 min at maximum speed. The upper phase was transferred to a new Eppendorf tube and the organic extraction was repeated. The upper phase was collected and precipitated using 50 µL 3M NaAc pH 5.6 and 1250 µL cold 100% ethanol for 30 min at -80°C. After centrifugation for 30 min at maximum speed, the DNA pellet was washed using cold 70% ethanol, dried in a speed vac and dissolved in 50µL nuclease-free water. For all samples, 260/280 ratios ranged between 1.7 and 1.9, and 260/230 ratios were higher than 1.6. A total of 33 DNA samples was prepared for Methylated DNA ImmunoPrecipitation (MeDIP)-chip analysis.

*MeDIP, whole genome amplification and methylation enrichment assessment:* Genomic DNA was fragmented to range between 200bp to 600bp using a Bioruptor, purified using silica columns (Zymo Research, Freiburg, Germany) and eluted in Tris-EDTA buffer pH 8.0. MeDIP was performed using the MagMeDIP kit (Diagenode, Liege, Belgium) according to the manufacturer’s protocol. IP incubation mix (containing magbuffer A, magbuffer B, 1.5 µg methylated DNA positive control, 1.5 µg unmethylated DNA negative control) was added to 1.2 µg sonicated sample and denatured at 95°C. A 10% aliquot was kept aside as Input sample and stored at 4°C. The remaining sample was immunoprecipitated with 5µL antibodymix (containing an antibody against 5’-methylcytidine (provided in the kit) magbuffer A and magbuffer C) and 20 µL magnetic beads. Immunoprecipitation was performed overnight at 4°C on a rotating wheel. The following day, magnetic beads were washed twice with wash buffer 1 and once with wash buffer 2 and kept on ice. DNA isolation was performed using the IPure kit (Diagenode, Liège, Belgium). Elution buffer was added to the MeDIP-bead pellet (50 µL) and to the Input sample (92.5 µL) and incubated for 15 min at room temperature on a rotating wheel. Supernatant was transferred to a new Eppendorf tube using a magnet. Samples were subsequently incubated with 2 µL of glycogen carrier, 100 µL 100% isopropanol and 15 µL magnetic beads and incubated for 1h at room temperature on a rotating wheel. Magnetic beads were washed with wash buffer 1 and wash buffer 2 for 5 min. The DNA was eluted twice with 75 µL buffer C and incubated for 15 min at room temperature on a rotating wheel. All samples were precipitated with sodium acetate and ethanol with glycogen as a carrier for 30 min at -80°C and centrifuged at max speed for 30 min at 4°C. The cell pellet was washed with 70% ethanol and after air drying resuspended in 10 µL MQ. Both Input and MeDIP samples were amplified by whole genome amplification (WGA) using the WGA2 kit (Sigma Aldrich) following the manufacturer’s instruction, without performing the fragmentation step. WGA reactions were cleaned up using silica columns (Sigma Aldrich) and eluted in water. Methylation enrichment in the paired samples MeDIP/Input was derived from qPCR data by calculating the ratio positive control/negative control, applying the ΔΔCq method using the primers included in the kit.

*MeDIP-Chip:* Whole genome analysis of DNA methylation levels was performed on Human 2.1M Deluxe Promoter Arrays (Roche NimbleGen, Basel, Switzerland). These arrays have a density of 2.1 million probes (50-75 oligonucleotides long, median probe spacing 100 bp) that represent all annotated human promoters (~ 26,210), 27,867 CpG islands and 750 miRNA promoters per slide. Labelling and hybridization of arrays was performed according to the manufacturer’s protocol. Briefly, Input and MeDIP DNA were labelled with Cy3 and Cy5, respectively, by random priming using the Dual Color DNA labelling kit (Roche NimbleGen) and hybridized with the NimbleGen hybridization kit (Roche, NimbleGen). Samples were hybridized overnight on the 2.1M Deluxe Promoter Arrays using the HX1 mixers and the NimbleGen Hybridization system 4. Slides were washed using the NimbleGen wash buffer kit and scanned using the 2 µm high resolution NimbleGen MS 200 micro array scanner.

Signal intensity data was extracted from the scanned images of each array using NimbleScan v2.6 software and quantile-normalized on a per-channel basis. Log2 ratios of the intensities were computed (ratio of MeDIP signal / Input signal) and for each array, centering was performed by subtracting the global array bi-weight mean of the log2 ratios such that the computed log2 ratios were centered around 0. Detection of differential methylation was performed using the Probe Sliding Window-ANOVA algorithm (PSW-ANOVA). PSW-ANOVA was implemented in the R statistical programming environment (v2.15.3) (<http://www.r-project.org>) as a custom script and was provided by Roche NimbleGen as previously described (van Breda et al. 2014). In short, PSW-ANOVA (sliding window of 750 bp comprising 7 probes and a FDR corrected p-value < 0.01) was used to identify differentially methylated regions (DMR) which were statistically significantly different between the different conditions tested in the experiment, i.e. treatment versus time-matched control. Peaks were identified in the DMR by searching for regions containing at least 8 significant consecutive probes (p<0.01). Peaks were mapped to promoter regions (from 3 kb upstream to 1 kb downstream of the transcription start site (Young et al. 2011) and CpG islands of genes using the NimbleScan v2.6 software. A control-corrected median log2 ratio was calculated for each gene. Log2 ratios > 0 indicate hypermethylation and log2 ratios < 0 indicate hypomethylation.

***Histone acetylation analyses using Chip-on-chip***

*Sample preparation:* Chromatin immunoprecipitation was performed using the SimpleChIP® Enzymatic Chromatin IP Kit (Magnetic Beads) (Cell Signaling Technology). After removal of the medium, ~5.10^6^ cells were cross-linked for exactly 10 min in the culture dish using 10 mL fresh medium containing 270 µL 37% formaldehyde (Merck) (1% end concentration) on a rocking platform at room temperature. Next, 1 mL 1.25M glycine was added and incubation was continued for exactly 5 min. Medium was discarded, and the cells were washed twice with ice-cold phosphate buffered saline (PBS, Sigma 79382). After removal of the PBS, 1.5 mL PBS/1 µM 0.1 M PMSF (Sigma P7626) were added and cells were collected by scraping with a rubber policeman and collected in an Eppendorf tube. Lysates were centrifuged for 5 min at 500 g at 4°C, and supernatant was discarded. Cell pellets were resuspended in 1 mL ice-cold Buffer A (0.5 µM DTT, Protease Inhibitor Cocktail (PIC), 1 µM PMSF), incubated for 10 min and regularly mixed. Lysates were then centrifuged for 5 min at 500 g at 4 °C. Supernatant was removed and pellets were resuspended in 1 mL Buffer B (0.5 µM DTT), incubated for 10 min and regularly mixed. The lysates were centrifuged for 5 min at 500 g at 4°C and resuspended in 1 mL Buffer B. Micrococcal Nuclease (1µL) was added for 20 min at 37°C, in order to digest the DNA to fragments of approximately 150 – 900 bp. Digestion was stopped with 100 µL 0.5M EDTA. DNA was pelleted by centrifugation for 1 min at maximum speed at 4 °C. Pellets were resuspended in 1 mL CHIP buffer containing PIC and 1 µM PMSF and incubated on ice for 10 min. Samples were sonicated in order to break the nuclear membrane. Lysates were clarified by centrifugation for 10 min at maximum speed at 4 °C. Supernatant containing the cross-linked chromatin was transferred to a new Eppendorf tube and 50 µL were used for analysis of chromatin digestion and concentration. Immunoprecipitation reactions were performed on 100 µL chromatine.

*Analysis of Chromatin Digestion and Concentration:* Chromatin samples (50 µL) were incubated with 100 µL nuclease-free water, 6 µL 5 M NaCl and 2 µL RNAse A for 30 min at 37°C. Next, 2 µL proteinase K were added and incubated for 2h at 65°C. DNA samples were purified using the DNA spin columns provided in the kit. After purification, DNA fragment size was determined by gel electrophoresis and DNA concentration was measured on a NanoDrop® ND-1000 spectrophotometer (Thermo Scientific). Fragments of DNA were between 150 and 900 bp and concentration was 50 – 200 µg/mL for all samples.

*Chromatin ImmunoPrecipitation (ChIP):* For ChIP, 400 µL CHIP buffer and 2 µL PIP were added to 100 µL chromatin sample. A 2% Input sample (10 µL) of the diluted chromatin was kept aside. For analysis of H3K9Ac, which is the classic histone mark for transcriptional activation (Berger 2007), 10 µL anti-Acetyl-Histone H3 (Lys 9) (C5B11) rabbit mAb #9649 antibody were added. In addition, histone H3 (D2B12) XP rabbit mAb #4620 was used as a positive control and normal rabbit IgG #2729 was used as a negative control. IP samples were incubated overnight at 4°C with rotation. The next day, 30 µL ChiP Grade Protein G Magnetic Beads were added and samples were incubated for 2h at 4°C with rotation. IP samples were then washed 3 times and Protein G Magnetic Beads were pelleted in a Magnetic Separation Rack. Supernatant was removed and low salt buffer with CHIP buffer was added to the Beads and incubated at 4°C for 5 min with rotation. After the last wash, 1 mL of high salt wash buffer (CHIP buffer, 70 µL NaCl) was added to the Beads and incubated at 4°C for 5 min with rotation. CHIP elution buffer (150 µL) was added to the 2% Input samples and kept aside. The Protein G Magnetic Beads containing the IP samples were pelleted in a Magnetic Separation Rack. Supernatant was removed and 150 µL CHIP elution buffer were added to each IP sample. Chromatin was eluted from the Protein G Magnetic Beads for 30 min at 65°C. The beads were pelleted and the eluted chromatin supernatants were transferred to a new Eppendorf tube. To all tubes, including the 2% Input samples, 6 µL 5 M NaCl and 2 µL proteinase K were added and incubated for 2h at 65°C. Samples were then purified using the DNA spin columns provided in the kit. Enrichment in the paired samples IP/Input was derived from qPCR data by calculating the positive control/negative control ratio, by applying the ΔΔCq method using the primers included in the kit.

*ChIP-chip, data analyses and selection of differentially acetylated genes:* H3K9 acetylation of human promoters was studied using the Human 2.1M Deluxe Promoter Array (Roche NimbleGen, Basel, Switzerland). Labelling, hybridization and washing of arrays was performed according to the manufacturer’s protocol as described in the MeDiP-chip section*.* Data analysis and selection of differentially acetylated genes was performed with the same workflow as for the MeDiP-chip data.
